# Books and Magazines

**Published:** 1913-08

**Authors:** 


### Books and Magazines.

LIPPINCOTT'S NEW MEDICAL SERIES.—TEXT-BOOK OF DISEASES OF THE NOSE, THROAT AND EAR.—For the use of students and General Practitioners. By Francis J. Packard, M. D., Professor of Diseases of the Nose and Throat in the Philadelphia Polyclinic Hospital and College for Graduates in Medicine; Aurist to the Out-patient Department of the Pennsylvania Hospital. Second edition, with 145 illustrations. Philadelphia and London: J. B. Lippincott Company, 1913. Price, $3.50.

This beauty is a luxury as well as a necessity. It has been but a short time since Dr. Packard launched the first edition of this splendid work, and it took like hot cakes. Here comes a second edition, made necessary already by the strides that have been made in the progress of laryngology and rhinology. These advances have been incorporated in the work before us. The sections dealing with diseases of the tonsils and adenoid growths have been rewritten as well as a description of the process employed in direct tracheobronchoscopy and aesophagoscopy.

THE PRACTICAL MEDICINE SERIES.—Comprising ten volumes on the year's progress in medicine and surgery. Under the general editorial charge of Gustavus P. Head, M. D., Professor of Laryngology and Rhinology, Chicago Post-Graduate Medical School, and Charles L. Mix, A. M., M. D., Professor of Physical Diagnosis in the Northwestern University Medical School.

Volume I, General Medicine, edited by Frank Billings, M. S., M. D., head of the Medical Department and Dean of the Faculty of Rush Medical College, Chicago, and J. H. Salisbury, A. M., M. D., Professor of Medicine, Chicago Clinical School. Series 1913. Year-Book Publishing Company, Chicago.

Volumes 1 and 2 are received. Volume 1 contains all that is new in the practice since this time last year, culled from the fleeting medical journals and put in permanent form.

Volume II, General Surgery, edited by John B. Murphy, A.

M., M. D., LL. D., Professor of Surgery in the Northwestern University; Attending Surgeon and Chief of Staff of Mercy Hospital, Wesley Hospital, St. Joseph's Hospital and Columbus Hospital, Consulting Surgeon to Cook County Hospital and Alexian Brothers Hospital, Chicago, Ill. Year-Book Publishing Co., Chicago, 1913. Price of this volume, $2. Price of the series of ten volumes, $10. They appear about every six weeks. Sold separately.

Murphy's volume on Surgery is eagerly awaited each year and is snapped up because it always contains the latest and newest in surgery.

---

THE NARCOTIC DRUG DISEASES AND ALLIED AILMENTS. By George E. Pettey, M. D. F. A. Davis Co., Publishers, Philadelphia. Price, $5.00 net.

In this volume narcotic addiction (including alcoholism) is treated as a disease—a toxemia—of drug, auto and intestinal origin, the management and treatment of which belong to the field of internal medicine and not to neurology.

Long standing serious errors of far-reaching consequences are corrected, the true pathology and pathogenesis clearly and fully shown, and as a natural corollary the most efficient treatment is completely set forth in these pages.

It is fundamentally different from any other work published in this heretofore baffling and obscure department of human medicine.

Dr. Pettey is the first author who, in my opinion, takes the correct view of the subject. The prevalent opinion amongst doctors is that all that is necessary is to "break the habit,"—to "withdraw the drug." There was never a greater mistake. There is no specific, no cure for drug and alcohol addiction. They are true psychoses, and require months; yea, years treatment. The whole system is poisoned and the will so weakened that a patient must be watched and cared for until he can resist the craving—the accustomed stimulant. Get this book. It will be a revelation to you.

---

Diseases of the Ear. By Philip D. Kerrison, M. D., Professor of Otology, New York Polyclinic Medical School and Hospital; Junior Aural Surgeon to the Manhattan Eye, Ear, Nose and Throat Hospital; Aural Surgeon to the Willard Parker Hospital for Infectious Diseases, and to the Polyclinic Hospital; member of the American Laryngological, Rhinological and Otological Society; of the American Otological Society, and of the New York Otological Society, and of the New York Academy of Medicine. 331 illustrations in text, and 2 full pages in color. Philadelphia and London: J. B. Lippincott Co., 1913. Price, $5.00.

I can not do better in a notice of this notable book—hot off the press—than to use some of the author's own words in the preface. He says that more notable advances have been made in otology in the last decade than in any other branches of medicine,—and proceeds to point them out as "a necessary consequence of the new light upon the syphilitic lesions of the labarynth and auditory nerve, resulting from the recent world-wide renewal of interest in the study of all phases of syphilis, and the investigations still in progress as to the influence of the autogenous vaccines and leucocyte extracts upon certain phases of aural disease.

In aural surgery our activities can no longer be confined to the narrow limits of the tympanum and mastoid process, but must include the more hazardous field of intra-cranial surgery, and the yet more delicate and difficult work upon the auditory labarynth itself. There is, then, some justification at the present time for yet another book; i. e., an attempt to present the complex subject of otology in the light of recent advances."

It seems to me that the above is all that is necessary to say in bringing this timely and most valuable book to the notice of our readers, unless I add that in the section devoted to operative surgery the plan of illustrating such successive steps of the various operations is an innovation which will be greatly appreciated, as one eyesight is worth two hearsays, and all of us like to be shown, the first time. I think highly of this book, and were I an aural surgeon I would not be without it at any price. The mechanical execution of the work is up to the Lippincott standard.

A Reference Hand-Book of the Medical Sciences.—Embracing the entire range of Scientific and Practical Medicine and Allied Science. By various writers. Third edition, completely revised and rewritten. Edited by Thomas Lathrop Stedman, A. M., M. D. Complete in eight imperial quarto volumes. Volume II. Wm. Wood & Co., New York. Complete in 8 volumes: Cloth, $56; leather, $64; half Morocco, $72.

This standard book of reference has been before the profession so long, and is so well known that a review of its contents would seem to be superfluous. But in bringing to the attention of my readers the second volume of the new edition (third), it is proper to point out that many changes and improvements have been made in order to bring the work fully abreast of the advanced state of medical science, as many and notable advances have been made since the appearance of the last edition. We reviewed Volume 1 of this new edition in a recent number, as did most of the medical journals, and the reviews for the most part were favorable and highly complimentary. This, the second volume, contains even a larger proportion of entirely rewritten articles and more new illustrations; and the great number of short (new) articles in this volume is a striking feature,—an innovation which I am sure will appeal to everybody. This work is as standard as Gray's Anatomy or the U. S. P., and is indispensable, as it has been revised and brought fully up to the present.

